# Nutritional status of school-age children - A scenario of urban slums in India

**DOI:** 10.1186/0778-7367-70-8

**Published:** 2012-04-17

**Authors:** Anurag Srivastava, Syed E Mahmood, Payal M Srivastava, Ved P Shrotriya, Bhushan Kumar

**Affiliations:** 1 Department of Community Medicine, Shri Ram Murti Smarak Institute of Medical Sciences, Bareilly (U.P.), India; 2 Department of Community Medicine, Rohilkhand Medical College and Hospital, Bareilly (U.P.), India; 3 G-48 Sanjay Gandhi Puram Faizabad Road, Lucknow (U.P.), India

**Keywords:** Growth monitoring, Malnutrition, School-age Children, Stunting, Wasting

## Abstract

**Background:**

One of the greatest problems for India is undernutrition among children. The country is still struggling with this problem. Malnutrition, the condition resulting from faulty nutrition, weakens the immune system and causes significant growth and cognitive delay. Growth assessment is the measurement that best defines the health and nutritional status of children, while also providing an indirect measurement of well-being for the entire population.

**Methods:**

A cross-sectional study, in which we explored nutritional status in school-age slum children and analyze factors associated with malnutrition with the help of a pre-designed and pre-tested questionnaire, anthropometric measurements and clinical examination from December 2010 to April 2011 in urban slums of Bareilly, Uttar-Pradesh (UP), India.

**Result:**

The mean height and weight of boys and girls in the study group was lower than the CDC 2000 (Centers for Disease Control and Prevention) standards in all age groups. Regarding nutritional status, prevalence of stunting and underweight was highest in age group 11 yrs to 13 yrs whereas prevalence of wasting was highest in age group 5 yrs to 7 yrs. Except refractive errors all illnesses are more common among girls, but this gender difference is statistically significant only for anemia and rickets. The risk of malnutrition was significantly higher among children living in joint families, children whose mother's education was [less than or equal to] 6th standard and children with working mothers.

**Conclusions:**

Most of the school-age slum children in our study had a poor nutritional status. Interventions such as skills-based nutrition education, fortification of food items, effective infection control, training of public healthcare workers and delivery of integrated programs are recommended.

## Background

School age is the active growing phase of childhood [1]. Primary school age is a dynamic period of physical growth as well as of mental development of the child. Research indicates that health problems due to miserable nutritional status in primary school-age children are among the most common causes of low school enrolment, high absenteeism, early dropout and unsatisfactory classroom performance. The present scenario of health and nutritional status of the school-age children in India is very unsatisfactory. The national family health survey (NFHS) data show that 53% of children in rural areas are underweight, and this varies across states. The percentage of underweight children in the country was 53.4 in 1992; it decreased to 45.8 in 1998 and rose again to 47 in 2006 [2].

Undernutrition in childhood was and is one of the reasons behind the high child mortality rates observed in developing countries. Chronic undernutrition in childhood is linked to slower cognitive development and serious health impairments later in life that reduce the quality of life of individuals. Nutritional status is an important index of this quality. In this respect, understanding the nutritional status of children has far-reaching implications for the better development of future generations.

Growth monitoring is universally used to assess nutritional status, health and development of individual children, and also to estimate overall nutritional status and health of populations. Compared to other health assessment tools, measuring child growth is a relatively inexpensive, easy to perform and non-invasive process.

Geographical relocation from rural areas to urban localities will expose migrants to new environmental challenges. Urban slum dwellers are exposed to poor environmental conditions (overcrowding, poor quality drinking water and sanitation, no removal of waste). Ignorance and difficult conditions of life in the slums are likely to result in improper food habits, low health care use and hygiene awareness and lack of knowledge of the origin of sickness and proper measures for the cure. The situation is further worsened due to lack of necessary health centers, medicines, and health care personnel. Children living under such conditions are at especially high risk for health and nutritional problems.

Anthropometric examination is an almost mandatory tool in any research to assess health and nutritional condition in childhood. Physical measurements like body weight, height, circumference of arm and calf, triceps skin fold of children have been extensively used to define health and nutritional status of communities. Based on the age, body weight and height, a number of indices such as height-for-age and weight-for-height have been suggested [3]. The children are classified using three categories: 'underweight' (low weight-for-age), 'stunting' (low height-for-age) or 'wasting' (low weight-for-height). Low anthropometric values are those more than 2 SD away from the CDC 2000 (Centers for Disease Control and Prevention) standards [3-5].

Stunting is defined as a low height-for-age for children, and it measures the past (chronic) child undernutrition. Children with z-scores < -2.00 are said to be stunted and those < -3.00 severely stunted.

Wasting is defined as low weight-for-height for children, and it is a measure of current or acute undernutrition. Children with z-scores < - 2.00 are said to be wasted.

Underweight is defined as low weight-for-age and it reflects past (chronic) and present (acute) undernutrition. Children with z-scores < -2.00 are said to be underweight.

The nutritional status of children does not only directly reflect the socioeconomic status of the family and social wellbeing of the community, but also the efficiency of the health care system, and the influence of the surrounding environment. The present study in selected slums of Bareilly City in the state of Uttar-Pradesh (UP), India, aimed to evaluate the overall prevalence of undernutrition, to assess age-sex trends in the level of undernutrition, to assess explaining factors and to recommend measures for correction of the nutritional deficit of the vulnerable population group and to provide baseline data for future research.

The objectives of the present study are:

1. To assess the prevalence of underweight, stunting, and wasting in children of 5 to 15 years old.

2. To analyze factors associated with malnutrition in children

## Methods

This cross-sectional study, in which we explored nutritional status in school-age slum children 5 to 15 years old, took place between December 2010 and April 2011 in urban slums of Bareilly (UP), India. The sample size of 384 was calculated assuming the prevalence of malnutrition was 50%, with relative precision of 10% at 95% confidence. For this study, 3 slums (Faltuganj, Kurramgotia and Kalibadi) were randomly selected from the urban area of the Bareilly district. All children aged 5-15 years from each of these slums were examined. A total of 512 children (297 boys and 215 girls) were interviewed and examined. A pre-designed and pre-tested questionnaire was used to interview the study participants to elicit information on family characteristics like residence, religion, type of family, education and occupation of parents; and information on individual characteristics like age, sex and eating habits. Anthropometric measurements were taken and noted by trained field workers. The questionnaire was pre-tested on 5 children from each slums. Necessary modifications were made in the questionnaire before the start of the study.

Ethical approval was obtained from Shri Ram Murti Smarak Institute of Medical Sciences, Bareilly (UP) Institution Review Board. For participation of the study subjects parents/guardians/caregivers were informed about the study objectives and gave informed written consent prior to inclusion into the study.

Each child's height and weight were measured in the metric system, using standardized technique recommended by Jelliffe [6]. A stadiometer (measuring rod) capable of measuring to an accuracy of 0.1 cm was used to assess height of the subjects. The subject was made to stand without footwear with the feet parallel and with heels, buttocks, shoulders, and occiput touching the measuring rod, hands hanging by the sides. The head was held comfortably upright with the top of the head making firm contact with the horizontal head piece. A portable balance with an accuracy of 100 g was used to record the weight of the subjects. Children were instructed to stand on the balance with light clothing and without footwear and with feet apart and looking straight. Weight was recorded to the nearest value.

Height for age (stunted), weight for height (wasted), and weight for age (under weight) for each child were calculated [3] and compared with the CDC 2000 [4]. Cut-off point values between ± 2 SD were considered normal [5].

Vitamin A deficiency was diagnosed by the presence of Bitot's spots and conjunctival xerosis. Rickets was diagnosed by abnormality in skeletal development, like knock-knees and bowed legs. Anaemia was diagnosed from clinical signs such as pallor of the conjunctiva/tongue.

After collection, all data were compiled and analyzed and appropriate statistical tests were applied. P < 0.05 was considered as statistically significant. Multivariate analysis was carried out, using the odds ratio (OR) to test for associations between various socio-economic indicators and nutritional status.

## Results

**Figure 1:**
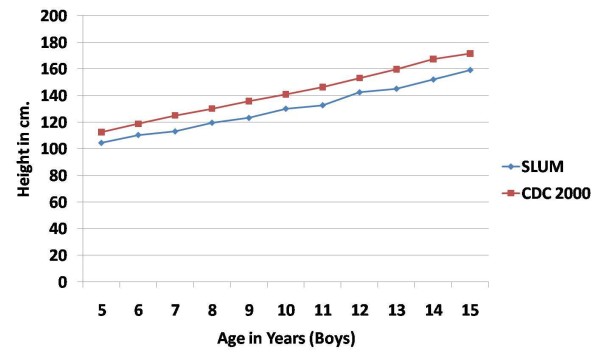
**Mean height (in cm) of school-age boys in urban slums of bareilly (UP), India (2010-2011) compared to the CDC 2000 reference**.

**Figure 2:**
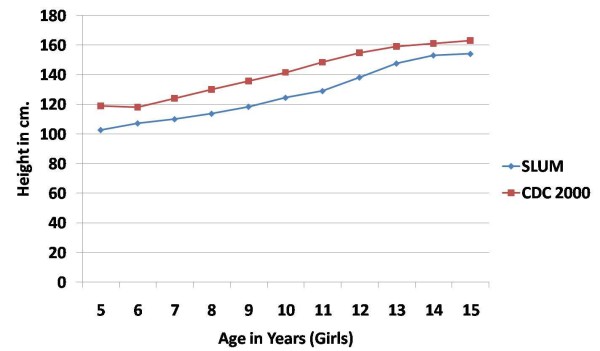
**Mean height (in cm) of school-age girls in urban slums of bareilly (UP), India (2010-2011) compared to the CDC 2000 reference**.

The mean height of girls was lower than that of the boys in all age groups except the 13-14 years old age group in which girls were taller than boys. This difference in height of boys and girls was not significant in any age group. The mean height of boys and girls of the study group was lower than the CDC 2000 standards in all age groups. (Figures 1 and 2)

**Figure 3:**
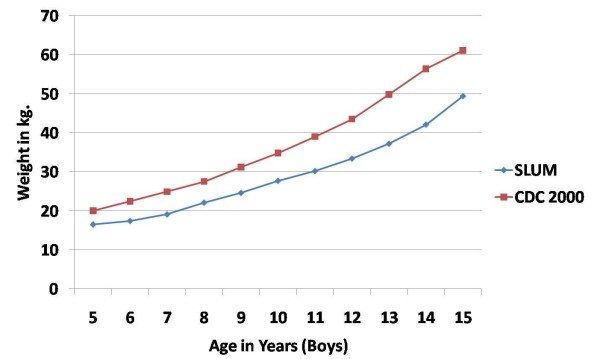
**Mean weight (in kg) of school-age boys in urban slums of bareilly (UP), India (2010-2011) compared to the CDC 2000 reference**.

**Figure 4:**
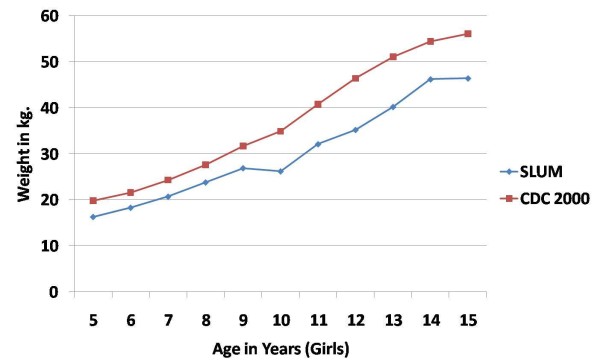
**Mean weight (in kg) of school-age girls in urban slums of bareilly (UP), India (2010-2011) compared to the CDC 2000 reference**.

The mean weight increased from 16.46 kg and 16.28 kg for boys and girls respectively in the 5 yr age group to 49.40 kg and 46.38 kg respectively in the 15 yr age group. The mean weight of girls was higher than the boys, in most of the age groups. However, there was no statistically significant difference in the mean weights of boys and girls in any of the age groups. In comparison with the CDC 2000 standard, the mean weight of boys and girls of the present study was found to be lower in all age groups. (Figures 3 and 4)

Regarding nutritional status, prevalence of stunting (long duration malnutrition) and underweight was found to be the highest in age group 5-6 yrs and 11-12 yrs respectively whereas maximum prevalence of wasting (short duration malnutrition) was found in age group 7-8 yrs. In all age groups most of malnourished children belonged to the underweight category. Among boys, 30.7% and 18.1% belonged to wasted and stunted nutritional status. 16.1% of girls belonged to stunted nutritional status indicating higher prevalence of long duration malnutrition among girls. Overall 33.3% of children were wasted whereas 18.5% were stunted and 46.8% were in normal nutritional status. The nutritional status was positively correlated to age indicating poor nutritional status of younger children. No significant association was found between gender and nutritional status of children. The results highlighted the higher prevalence of malnutrition among younger children; therefore, younger age groups should be the main target for nutritional surveillance and interventions (Table 1).

**Table 1:** The nutritional status of school-age children by age and gender in urban slums of bareilly (UP), India 2010-2011

Age (in years)	Nutritional Status (%)				Total
	
	Normal	Underweight (low weight for age)	Wasted(SDM) (low weight for height)	Stunted(LDM) (low height for age)	
**5-6**	50 (45.9)	39 (35.8)	34 (31.2)	25 (22.9)	109

**7-8**	64 (46.7)	55 (40.1)	49 (35.8)	24 (17.5)	137

**9-10**	48 (48.5)	35 (35.4)	31 (31.3)	20 (20.2)	99

**11-12**	30 (44.1)	28 (41.2)	23 (33.8)	15 (22.1)	68

**13-15**	53 (48.2)	44 (40.0)	37 (33.6)	20 (18.2)	110

**Gender**					

**Boys**	157 (51.6)	102 (33.6)	92 (30.7)	55 (18.1)	304

**Girls**	88 (40.2)	99 (45.2)	82 (37.4)	49 (22.4)	219

**Overall**	245 (46.8)	201 (38.4)	174 (33.3)	104 (19.9)	523

Except refractive errors, all other illnesses are more common among girls than boys, but this gender difference is statistically significant only for anemia. The most common illness found was anemia with prevalence of 37.5% followed by dental carries (18.5%) and throat infection (14.9%) (Table 2).

**Table 2:** The prevalence of nutritional disorders among school-age children by gender in urban slums of bareilly (UP), India 2010-2011 (Multiple Responses)

Nutritional disorders	Boys (n = 304)		Girls (n = 219)		Total (%)
		
	**No**.	%	**No**.	%	
Anemia	102	33.7	94	42.8	196 (37.5)
		
	**X^2 ^= 4.76, p = 0.0290**				

Vit A deficiency disorders	7	2.3	11	5.0	18 (3.4)
		
	**X^2 ^= 2.83, p = 0.0923**				

Refractive errors	27	8.9	14	6.3	41 (7.8)
		
	**X^2 ^= 1.09, p = 0.2962**				

Rickets	0	0.0	4	1.8	4 (0.8)
		
	**X^2 ^= 3.45, p = 0.06333**				

Dental caries	52	17.2	45	20.4	97 (18.5)
		
	**X^2 ^= 0.99, p = 0.3176**				

CSOM	2	0.7	2	0.9	4 (0.8)
		
	**X^2 ^= 0.03, p = 0.8580**				

Throat infections	42	13.7	36	16.2	78 (14.9)
		
	**X^2 ^= 0.69, p = 0.4062**				

Skin diseases	8	2.7	7	3.1	15 (2.9)
		
	**X^2 ^= 0.14, p = 0.7024**				

Univariate analysis showed a significantly higher risk of malnutrition among female children, children living in joint families, children with birth order > 2, children who were never breastfed, children whose father and/or mother had a low educational attainment (< 6th standard), children whose mother had a service/business. This implies the importance of the family characteristics in the causation or predisposition of an individual to malnutrition (Table 3).

**Table 3:** Univariate association of socio-economic factors with the malnutrition status of school-age children in urban slums of bareilly (UP), India, 2010-2011

*Variable*	*Total*	*Malnourished*	*OR(95% CI)*
***Sex of child***			

Male	304	147	1

Female	219	131	*1.59(1.12-2.26)*

***Type of family***			

Nuclear	143	39	*1*

*Joint*	380	239	4.52(2.96-6.90)

***Birth order***			

≤2	198	69	1

> 2	325	209	3.37(2.33-4.88)

***Ever breastfed***			

Yes	407	200	1

No	116	78	2.12(1.38-3.28)

***Mother's education***			

> 6^th ^standard	324	125	1

≤ 6^th ^standard	199	152	5.15(3.46-7.65)

***Father's education***			

> 6^th ^standard	412	207	1

≤ 6^th ^standard	111	71	1.76(1.14-2.71)

***Mother's occupation***			

Nonworking	278	94	1

Working	245	184	6.10(4.16-8.97)

***Father's occupation***			

Service/business	338	153	1

Laborers	185	125	2.52(1.73-3.67)

Step-down multiple logistic regression using backward LR method was applied to determine the significant correlates of malnutrition in the study population. The final model showed that joint family, birth order > 2, mother's education ≤ 6th standard and mother's occupation were significantly associated with malnutrition among the study population (Table 4).

**Table 4:** Multivariate association of socio-economic factors with the malnutrition status of school-age children in urban slums of bareilly (UP), India, 2010-2011

Variable	Odds ratio	95% CI
Joint family	4.03	2.41-6.18

> 2 birth order	3.09	2.16-4.19

Mother's education ≤ 6^th ^standard	3.81	2.37-5.96

Mother working	4.47	3.04-6.98

## Discussion

Children in the age group of 5-14 years are often considered as school-age. Since 1972, the United Nations Educational Scientific and Cultural Organization (UNESCO) considers 6-11 years as primary school age and 12-17 years as secondary school age for statistical purposes. In it is recorded that in India one fifth of the population consists of children between 5 and 14 years, which includes the primary and secondary school age. School age is considered as a dynamic period of growth and development because children undergo physical, mental, emotional and social changes. In other words the foundations of good health and sound mind are laid during the school age period. Hence the present study was formulated with the objective, to assess and find the major socio-economic correlates of nutritional status in school-age children.

The present study showed a growth lag in the basic parameters of height and weight as compared to the reference standards laid down by CDC 2000. Our findings are similar to that reported by other workers from India [7,8]. Best C. et al. also reported that underweight and thinness were most prominent in populations from South-East Asia and Africa, whereas in Latin America, the prevalence of underweight or thinness was generally below 10% [9].

Throughout the developing world, children fail to grow in length and weight in a remarkably similar age-specific pattern, despite vast differences in the prevalence of low weight (wt)/age and height (ht)/age between the regions [2]. We analyzed the prevalence of stunting, wasting and underweight as markers of undernutrition and our findings were similar as in South Africa, where stunting and underweight remain a public health problem in children, with a prevalence of 20% stunting and almost 10% underweight [10]. The anthropometric results of a study in Qwa Qwa also indicated that 2.8% of the total group of respondents was severely stunted, and that 11.3% were stunted [11].

Thus the differences in the degree of growth failure in weight and height have implications for assessing the true prevalence of chronic malnutrition. This is also important for monitoring trends or evaluating the effects of interventions [12]. There is a need to shift the focus from wt/age to ht/age and wt/ht for assessing malnutrition and identifying populations that could benefit from interventions.

The school children in the present study were found to be better nourished than the rural Punjab school children as reported in another recent study [13], where the prevalence of malnutrition was 87.4%. However, the standards of nutrition among children in the present study were lower than those found in children in Delhi by Dhingra et al. [14] and in urban school-age children in Tirupati as reported by Indirabai et al. [15]. Goyal et al. [16] found malnutrition among Ahmednagar school children to be 20% only, with 6.8% having severe malnutrition, which is much lower than rural school children of Punjab (37.6%) [13] and amongst school children of Madras, as found by Sunderam et al. (32.6%) [17]. These disparities in findings of different studies may be due to differences in study settings. The rate of undernutrition of the present study is quite similar to the findings of Medhi et al. [18] who recorded a prevalence rate of undernutrition of 53.9% among school-age children in Assam-India.

The evidence suggests that boys are more likely to be stunted and underweight than girls, and in some countries, more likely to be wasted than girls [19,20], but in the present study, undernutrition was significantly more prevalent in girls than boys. A number of studies in Africa suggest that rates of malnutrition among boys are consistently higher than among girls. Studies conducted in Ecuador [21] and in Tanzania [22] show that boys were more commonly affected than girls. One of the largest studies [20] of anthropometric status of rural school children in low income countries (Ghana, Tanzania, Indonesia, Vietnam and India) found the overall prevalence of stunting and underweight to be high in all five countries, ranging from 48 to 56% for stunting and from 34 to 62% for underweight. Boys in most countries tended to be more stunted than girls and in all countries, boys were more underweight than girls. These disparities in findings are due to differences in study frame, family setups, gender bias and parental preferences for male children in the Indian society.

Anemia was detected in 37.5% of children in the present study, which was more than in the children of rural school children in Punjab (22.5%) [13]. The prevalence of anemia in girls (42.8%) was significantly higher than in boys (33.7%). In our study diagnosis of anemia was exclusively based on clinical examination; no laboratory examination was done. Hence there is a possibility of underreporting of prevalence of anemia in this study population and this underreporting may be higher in boys. Prevalence of dental caries in the present study was higher than in rural Punjab school children (11.1%) [13], almost equal to the findings in Tirupati (20.9%) [15] and less than in Madras school children (38.6%) [17]. Gender differences observed in the prevalence of dental caries were statistically not significant.

Women's educational and social status, food availability, and access to safe water are well reported important underlying determinants that directly or indirectly cause malnutrition among children [23]. In our study mother's education was found to be a strong predictor of child nutritional status. Data analysis of National Family Health Survey (NFHS) 1 also showed that mother's education has a strong independent effect on a child's nutritional status even after controlling for the potentially confounding effects of other demographic and socioeconomic variables [24].

Earlier studies using household-level data have found mother's education to be positively associated with a number of measures of child health and nutritional status [25-31]. Results pointing to the importance of socioeconomic status indicators such as mother's education to children's nutritional status are consistent with findings in Yip et al. [32].

Further improvement in nutritional status with maternal education has been reported by other authors [33-36]. The pattern of declining incidence of stunting by mother's education in Cambodia is consistent with patterns observed in many other developing countries [37]. The pattern for wasting concurs with arguments found in several other studies [38,39] that wasting is influenced less by maternal characteristics than is stunting. One explanation is that mother's education has a limited effect on preventing illness such as diarrhea when there are widespread sources of infection.

Various studies have concluded that parental education, especially mothers' education, is a key element in improving children's nutritional status [40,41].

In the present study family type was significantly associated with all three indices of malnutrition. Similar results have been reported by Gopaldas et al. [34]. NFHS 1 survey also showed that children living in joint family setup were more likely to suffer chronic malnutrition than children from nuclear families. The results are different from a study by Singh [42] on children of urban slums as in their study > 70% of the families were nuclear.

It was clearly shown that children who had never been breastfed were at much higher risk of poor nutritional status. Thus breastfeeding is positive health behavior in this population, and should be encouraged.

One of the strongest predictors of malnutrition in this analysis was mother's working status. Children of nonworking mothers have better nutritional status than children of working mothers, possibly due to more time for caring of children [34,35]. Hence the busy time schedule of working mothers adversely affects the nutritional status of children. The NFHS II also observed a higher prevalence of these three indices of malnutrition in children of working mothers.

This study shows that maternal educational status, mother's working status and family type are important determinants of the nutritional status of the child. Efforts directed towards improvement of female literacy, women empowerment and restricting family size will have a positive impact on the nutritional status of school children.

## Conclusions

It is clear that the problem of malnutrition in India is of alarming magnitude, but also of great intricacy. The prevalence of underweight is among the highest in the world, nearly double that in Sub-Saharan Africa, and the pace of improvement lags behind what might be expected given India's economic growth. A major part of this problem is contributed by slum population.

Tackling malnutrition in urban slums requires a holistic approach, especially when targeting populations of school-age children. For effective implementation of this approach in urban slums following interventions are recommended.

### 1. Skills-based nutrition education for the family

Nutrition education should address family as a whole and not just the women. Nutrition education should focus on communication for behavioral change. The nutrition-related activities need to be based on qualitative research that has identified cultural and institutional constraints to good nutrition, detrimental attitudes and practices toward food and eating behavior. With creative thinking, nutrition and health-related activities can be incorporated into group activities, but needs to be perceived to be relevant to their lifestyles rather than imposed.

### 2. Fortification of food items

Any food commodity, be it sugar, milk, pulses, rice or condiments can be fortified with micronutrients.

### 3. Effective infection control

In slum environments, children are especially susceptible to a host of diseases and infections that compromise their health and immunity and, in turn, their nutritional status. Malnutrition and childhood diseases are interconnected and mutually reinforce one another. It is therefore extremely important that childhood diseases are identified, and appropriately treated, to contain the effect of the disease on child health.

### 4. Training public healthcare workers

Service providers should be equipped with knowledge and skills to implement a nutrition program efficiently. Appropriate training methodologies and tools need to be developed to train the service providers. Trained community link workers do not only enhance access to healthcare for the entire community but also deliver healthcare services and education to mothers and children where the public healthcare system is absent.

### 5. Deliver integrated programs

Intersectoral collaboration is recognized as one of the strategies to address problems of malnutrition. Nutrition education can have a significant effect in promoting healthy eating habits, and schools can contribute to reduce nutrition-related problems by integrating nutrition interventions into a comprehensive school health program.

## Competing interests

The authors declare that they have no competing interests.

## Authors' contributions

The authors' responsibilities were as follows: SA concieved the idea of this study, supervised the study, participated in the design of the research instrument, reviewed related literature, and participated in discussing findings and making recommendations on the basis of the findings of the study. He finalized the manuscript for submission. MSE concieved the idea of this study, participated in the design of the study, and had the major responsibility of coordinating the data collection. MSP participated in design of the work, analysis of the data and interpretation of the results. She also actively participated in the write-up of the study. SVP participated in design of the work, interpretation of data and writing of the manuscript, KB participated in data collection, study subjects management and manuscript writing. All authors have read and approved the final manuscript.
